# Isavuconazole and Liposomal Amphotericin B as Successful Combination Therapy of Refractory Invasive Candidiasis in a Liver Transplant Recipient: A Case Report and Literature Review

**DOI:** 10.1007/s11046-021-00599-1

**Published:** 2021-10-31

**Authors:** Georgios Odysseos, Ulrich Mayr, Gabor Bozsaki, Christian Seidensticker, Ursula Ehmer, Roland M. Schmid, Tobias Lahmer, Veronika Dill

**Affiliations:** 1 Klinik und Poliklinik für Innere Medizin II, Klinikum rechts der Isar der Technischen Universität München, Munich, Germany; 2 Klinik und Poliklinik für Innere Medizin III, Klinikum rechts der Isar der Technischen Universität München, Ismaninger Straße 22, 81675 Munich, Germany

**Keywords:** Candidemia, Invasive candidiasis, Liver transplantation, Antifungal combination therapy, Isavuconazole, Liposomal amphotericin B

## Abstract

Invasive fungal infections in liver transplant recipients are associated with elevated morbidity and mortality and pose a challenge to the treating physicians. Despite of lacking clinical data, the use of antifungal combination therapy is often considered to improve response rates in an immunocompromised patient population. We herein report a case of refractory invasive candidiasis in a liver transplant recipient treated successfully with a combination of isavuconazole und high-dose liposomal amphotericin B. The antimycotic combination treatment was able to clear a bloodstream infection with *C. glabrata* and led to regression of bilomas among tolerable side effects. The use of the above-mentioned antifungal combination therapy in a liver transplant recipient has not been reported previously. This case highlights the efficacy and safety of antifungal combination therapy in immunocompromised patients with refractory invasive candidiasis.

## Introduction

Liver transplantation (LT) is a well-established clinical procedure for the treatment of terminal liver diseases. Survival rates after LT have improved significantly over the past decades, reaching 85% at 1 year and 73% at 5 years considering the last 10-year period [1]. This accomplishment is mainly achieved by the introduction of new immunosuppressive agents, the improvement of surgical techniques and the early diagnosis and treatment of complications [2]. Irrespective of the administration of an antifungal prophylaxis, invasive fungal infections (IFI) can be observed in approximately 5–7% of liver transplant recipients [3, 4]. Previous studies reported that IFI in liver transplant recipients occurred mainly in the early phase after LT [5] and frequently in patients with renal insufficiency, vascular/biliary complications and re-transplantation [6]. Invasive candidiasis represents the most common IFI subtype and refers to candidemia, a bloodstream infection with *Candida* species (spp.) as well as deep-seated infection, with or without candidemia [7].

After diagnosis of candidemia antifungal therapy should be initiated immediately with an appropriate antifungal agent from the substance groups of azoles, echinocandins and liposomal amphotericin B. Despite proper therapy, candidemia causes significantly elevated morbidity and mortality in this immunocompromised patient population [6]. Consequently, IFI remain a challenging situation for the treating physicians and the use of antifungal combination therapy is often considered to improve response rates. Nevertheless, there is only limited clinical data available to support antifungal combination therapy over single-agent treatment in patients with invasive candidiasis [8]. Moreover, the usage of antimycotic combination treatment has never been evaluated as first-line or salvage therapy in liver transplant recipients [9]. To the best of our knowledge, this is the first report describing the successful combination of isavuconazole (ISA) and liposomal amphotericin B (AMB) in a liver transplant recipient with refractory invasive candidiasis.

## Case Report

A 52-year old female, who underwent LT past 3 months due to primary biliary cirrhosis, was admitted because of fever up to 40 °C. In the past history, short term after LT, increased cholestasis parameters and transaminases had been noted. Endoscopic retrograde cholangiography (ERC) had demonstrated ischemic-type biliary lesions, characterized by intrahepatic bile duct strictures, in the absence of perfusion restrictions. At that time a biliary cannulation had been performed. Post intervention both liver and cholestasis parameters had decreased. An acute rejection of the transplanted liver had been ruled out by biopsy. The post-transplant immunosuppression consisted of mycophenolate mofetil (MMF) and tacrolimus. Due to the absence of high-risk factors for fungal infections [5, 6], antifungal prophylaxis had not been administered post-transplant.

The physical examination on the day of admission to our hospital was unremarkable. The patient was hemodynamically stable, had normal breathing sounds, no cardiac murmur and the abdomen reconciled normal. Blood results showed high inflammation markers with elevated CRP, procalcitonin and IL-6 as well as marginally elevated transaminases with normal cholestasis parameters. An abdominal ultrasound examination showed slightly dilated peripheral bile ducts in the absence of focal lesion. Therapeutic ERC with double pigtail stent replacement was performed. Two of three blood cultures from admission day detected colonies of Enterococcus faecalis and Enterococcus casseliflavus. With similar detection of both bacteria in the bile culture, a biliary focus seemed very likely. Broad spectrum antibiotics with meropenem and linezolid were given. During hospitalization the patient developed infection-associated progressive neutropenia (neutrophil nadir 0.74 G/l on day 4). As a consequence, MMF was paused and immunosuppression was adapted with tacrolimus and prednisolone (short-term administration of a maximum of 20 mg prednisolone per day and gradual reduction to a maintenance dose of 5 mg per day). Of note, leukocytes increased within a week after drug discontinuation.

**Fig. 1:**
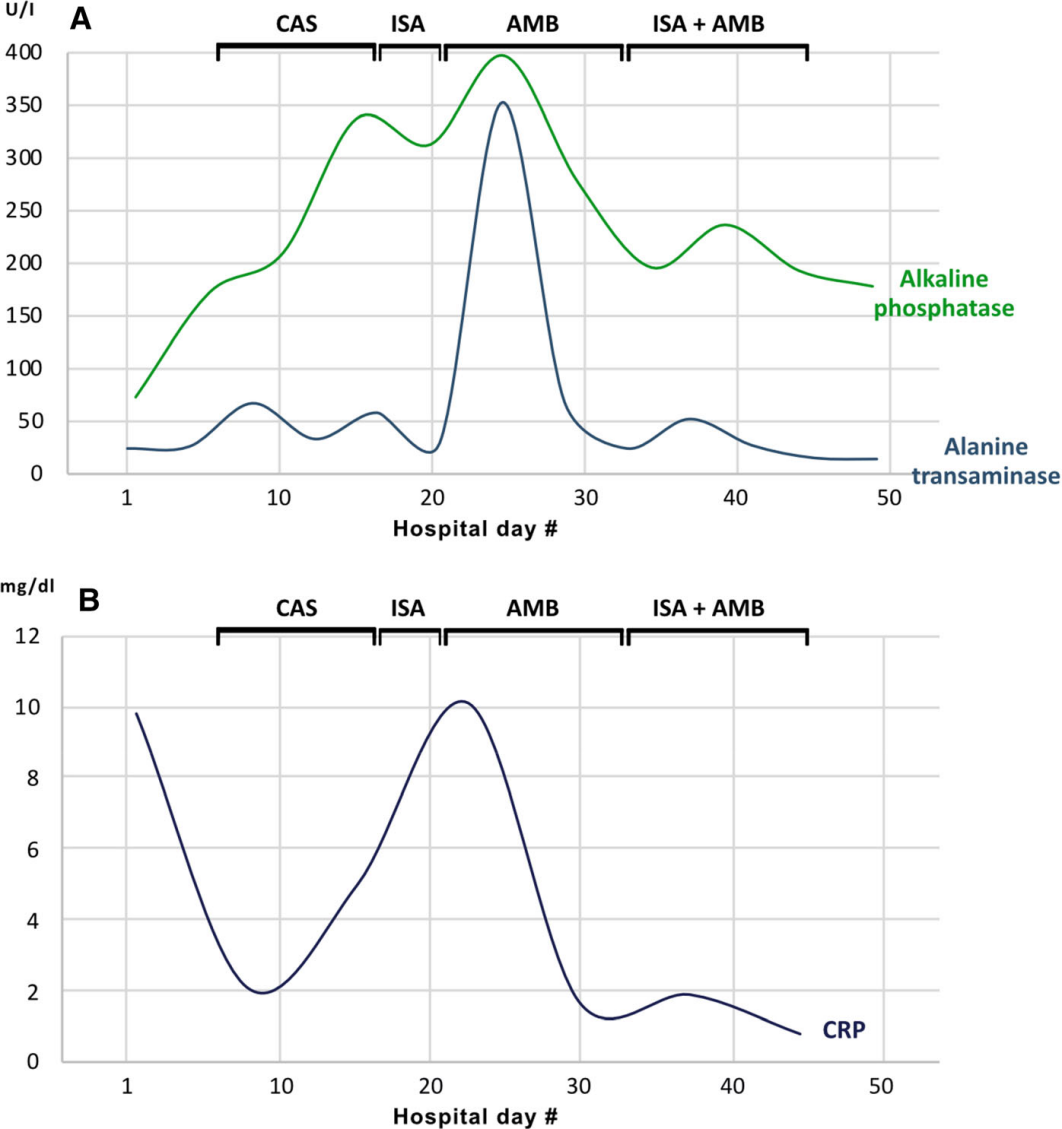
Longitudinal changes of alkaline phosphatase, alanine transaminase (**A**) and the inflammation marker C-reactive protein (CRP) (**B**) during the patient’s hospitalization. The antifungal agents used in the certain period are marked accordingly (CAS: caspofungin, ISA: isavuconazole, AMB: liposomal amphotericin B). Normal ranges for laboratory values: Alkaline phosphatase 40–129 U/I, alanine transaminase 10–50 U/I, CRP < 0,5 mg/dl

Systemic candidiasis was diagnosed for the first time in the follow-up blood cultures on hospital day 8 based on the evidence of *C. glabrata* (Table 1A). *Candida* sepsis was diagnosed due to organ dysfunction with a Sequential Organ Failure Assessment (SOFA) score > 2 points. Shortly after the diagnosis, an intravenous antifungal therapy with caspofungin (CAS; 70 mg loading dose on day 1, followed by 50 mg daily thereafter) was initiated. A biloma in liver segment VI/VII was detected for the first time on day 10 by abdominal sonography. Laboratory findings consisted of slightly increasing transaminases (Fig. 1). An endophthalmitis was ruled out within a week after diagnosis. Extensive imaging, including echocardiography, orthopantomography and magnetic resonance imaging (MRI) of the spine did not reveal any evidence of an infection focus with *C. glabrata*. The peripheral venous catheter was changed regularly. Nevertheless, blood cultures, repeatedly taken every 24–48 h, remained positive for *C. glabrata*.

**Table 1:** Antimycotic sensitivity testing for *C. glabrata* categorized as susceptible (S) or susceptible at increased exposure (IE) towards the indicated antifungal agents (A) on hospital day 8 and (B) on hospital day 26. Minimum inhibitory concentration (MIC) breakpoints (mg/L) were determined according to the recommendations of the European Committee on Antimicrobial Susceptibility Testing (EUCAST)

Antifungal agent	(A) Sensitivity testing on day 8	(B) Sensitivity testing on day 26
Amphotericin B	S (≤ 0,25 mg/L)	S (1 mg/L)
Voriconazol	IE (0,25 mg/L)	IE (0,25 mg/L)
Caspofungin	S (≤ 0,12 mg/L)	S (0,5 mg/L)
Flucytosin	IE (≤ 1 mg/L)	IE (≤ 1 mg/L)
Micafungin	S (≤ 0,06 mg/L)	S (≤ 0,06 mg/L)

**Fig. 2:**
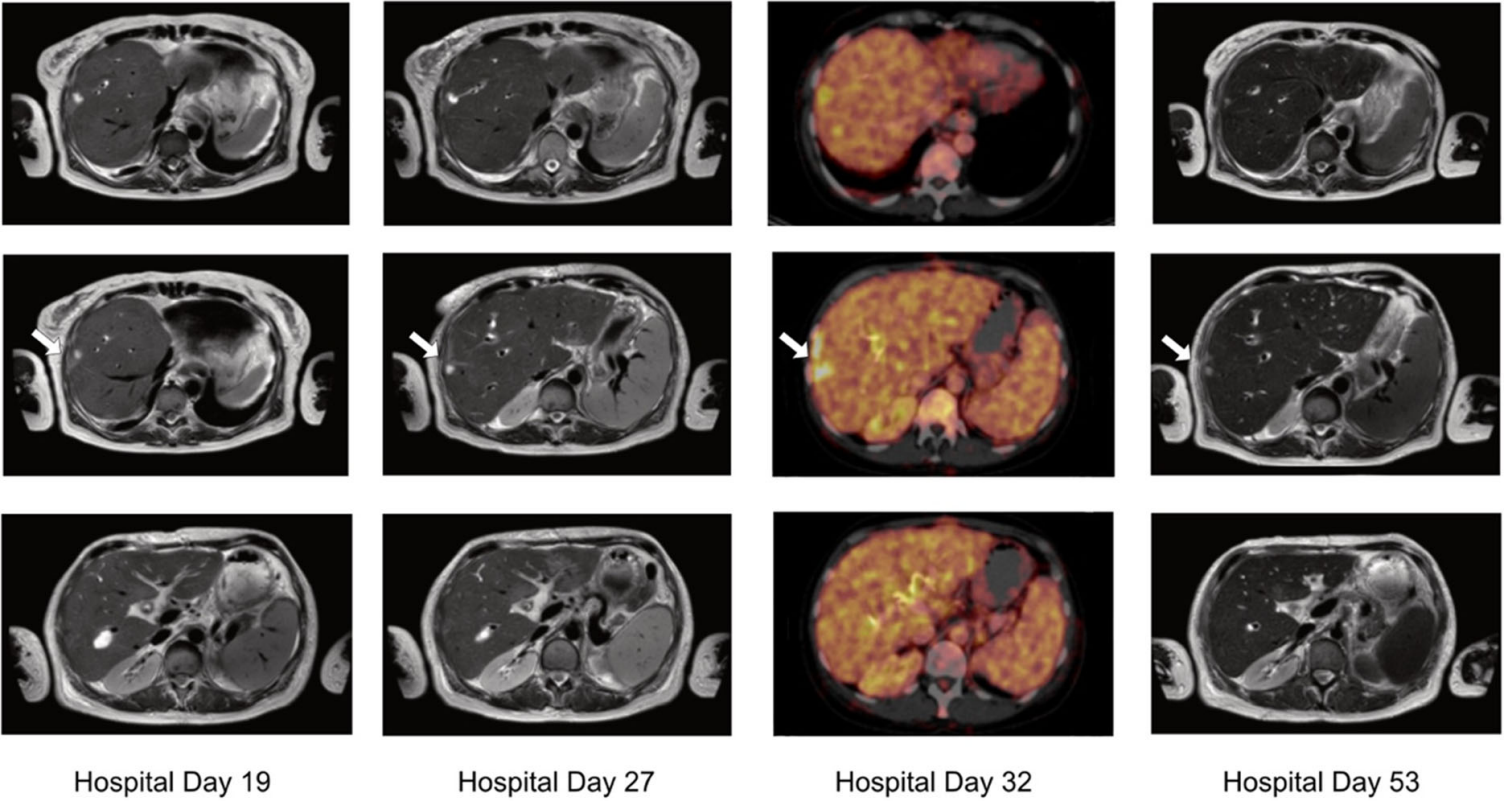
Image-based morphological monitoring of bilomas during the patient’s hospitalization at day 19 (MRI), day 27 (MRI), day 32 (PET/CT) and day 53 (MRI)

Due to persistent fungemia and recurrent episodes of fever, the intravenous antimycotic therapy was converted to isavuconazole (ISA; 200 mg three-times daily for two days, followed by 200 mg once daily) on hospital day 18. This substance was chosen contrary to other triazoles due to increased creatinine levels and hepatic enzymes during candidemia. Shortly after switching to isavuconazole, both aspartate aminotransferase (AST) and alanine aminotransferase (ALT) decreased (Fig. 1). However, further MRI imaging on hospital day 19 showed new bilomas with a maximum size of 23,8 × 15,2 mm (Fig. 2) in liver segments VII und VIII and an increasing expansion of bile ducts. Cholestasis was thoroughly reassessed through another ERC on day 21 demonstrating anastomotic stricture and intrahepatic stenosis. Because of complicated structural conditions in the transplanted liver, stent replacement was not possible. Bile drainage was optimized by implantation of three new stents. A percutaneous transhepatic biliary drainage was not required, since internal biliary drainage was possible through an ERC [10].

With persisting positive blood cultures and high systemic inflammation markers (Fig. 1), the antifungal therapy was changed to liposomal amphotericin B (AMB) on hospital day 22 at a daily dose of 3 mg/kg body weight. Nevertheless, candidemia perseverated despite test-regulated therapy (Table 1B). A follow-up MRI scan of the liver revealed size-progressive dimensioning of bilomas in liver segment VIII with also increasing changes in local liver parenchyma. Increased metabolic activity of the known lesions in liver segment VIII were demonstrated by positron emission tomography-computed tomography (PET/CT), consistent with ascending cholangitis (Fig. 2). There was no evidence of any further metabolically active sites. Due to the complex anatomical conditions of the transplanted liver, further intervention by ERC was decided to be forgone.

Subsequently we initiated an intensified dual antifungal therapy with high doses of liposomal amphotericin B (daily dose of 6 mg/kg of body weight) and isavuconazole (200 mg three-times daily for two days, followed by 200 mg once daily) on hospital day 34. From the following day on, all taken blood cultures remained sterile. Interestingly, liver enzymes decreased after starting dual therapy and despite the increased doses of liposomal amphotericin B in the combination regimen compared to monotherapy (Fig. 1). The intravenous antifungal combination therapy was continued for 14 days after the first negative blood culture and stopped afterwards. Six days after completion of therapy, a follow-up MRI scan of the liver was performed, where cholangitis and bilomas showed to be regressed (Fig. 2). It should be noted that during the course of the infection, creatinine levels increased, but remained stable with absence of oliguria or hyperkalemia during the further course of hospitalization.

**Fig. 3:**
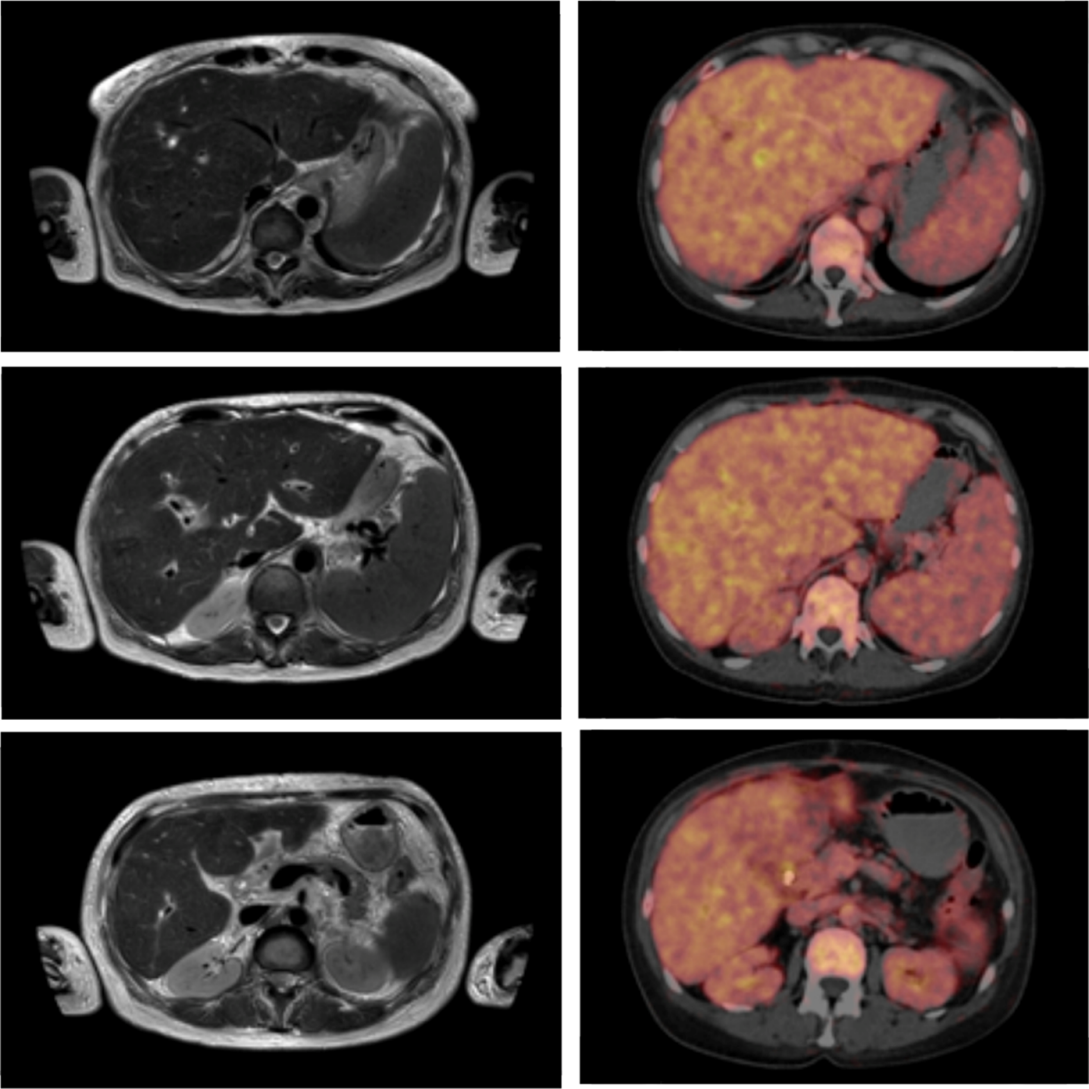
Follow-up imaging by MRI and PET/CT six weeks after discharge

The patient was hemodynamically stable during the entire stay at our normal ward and discharged without any further complication. Ten days after discontinuation of dual antifungal therapy an antifungal prophylaxis was administered with posaconazole (300 mg twice on day 1, followed by 300 mg once a day for five weeks), since this substance has been shown to provide high quality evidence in preventing IFI among immunocompromised patients [11]. Follow-up imaging was performed six weeks after discharge and showed further regression of bilomas compared to the previous examination (Fig. 3). A PET/CT demonstrated lacking metabolic activity of the formerly hypermetabolic sites, consistent with a reduced inflammatory activity. Furthermore, there was no evidence of *C. glabrata* in the follow-up blood cultures. Of note, creatinine levels had decreased subsequently and were only slightly elevated at the follow-up visit.

## Discussion

The reported case represents a typical example for IFI in immunocompromised patients. IFI occur in approximately 5–7% of liver transplant recipients [4] and are predominantly caused by infections with *Candida* spp. during early post-LT period [6]. With a mortality rate of 30.9% invasive candidiasis is a life-threatening complication in liver transplant recipients [12] and a demanding task for clinicians.

After diagnosis of candidemia it is essential to immediately initiate antifungal therapy. Treatment with an echinocandin (e.g., caspofungin: loading dose 70 mg, then 50 mg daily) is considered first-line therapy. These substances offer a favorable safety profile, good tolerability and early fungicidal activity, especially in regard to the recent development of azole-resistant *Candida* spp. [8]. Fluconazole can be used instead of an echinocandin in selected patients, including non-neutropenic patients who are not critically ill [8]. Supplementary, in patients with decompensated liver disease or high-risk liver transplant with proven or probable IFI, treatment with anidulafungin demonstrated efficacy and well-tolerated side effects [13]. In patients with azole- and echinocandin-resistant *Candida* infections, liposomal amphotericin B (3–5 mg/kg daily) can be used alternatively [8]. Of note, this antifungal agent caused more infusion-related events and nephrotoxicity than caspofungin [14]. In our patient isavuconazole, a new extended-spectrum triazole, was used because of its favorable tolerability profile, less concerns for nephrotoxicity and similar activity against most *Candida* spp. when compared to other azoles [8, 15–17]. The ACTIVE trial did not show non-inferiority of isavuconazole to caspofungin for first-line treatment of invasive candidiasis [18]. Isavuconazole is approved by the European Medicines Agency (EMA) and the U.S. Food and Drug Administration (FDA) for the treatment of invasive aspergillosis and invasive mucormycosis [17, 19–21]. In general, antimycotic therapy should be continued with a minimum duration of two weeks after documented clearance in the bloodstream [8]. Besides, an adjustment of immunosuppression is recommended [22].

The elevated mortality rate of IFI in immunocompromised patients has led to significant clinical interest in antifungal combination therapy [9]. Due to synergistic interaction between antifungal drugs, antimycotic combination therapy offers a wide spectrum of antifungal activity. Moreover, lower doses of application might reduce the development of resistance. Although previous studies have shown a tolerable side effect profile of dual antifungal therapy [23], such therapy remains controversial [23, 24]. For the purpose of this case, we are going to focus on publications and evidence for the use of antifungal combination therapy in candidemia and invasive candidiasis. A randomized and blinded multicenter trial from Rex et al. showed that the combination of fluconazole and liposomal amphotericin B was not antagonistic compared to fluconazole alone, and the combination trended toward improved success and faster clearance from the bloodstream infection with *Candida* spp. [25]. An older randomized study from Abele-Horn et al*.* demonstrated that the treatment of systemic *Candida* infections with amphotericin B and 5-flucytosine in intensive care patients was more effective than fluconazole alone [26]. It should be mentioned that in this study monotherapy with fluconazole was associated with less toxicity than combined therapy. In one report, fluconazole-resistant oropharyngeal candidiasis was successfully treated with a combination of fluconazole and terbinafine [27].

On the contrary, in vitro combination of isavuconazole and amphotericin B was consistently antagonistic against *C. glabrata* [28]. The triazole-polyene antagonism may be understood by modification of the ergosterol target by the azoles, decreasing amphotericin B activity [24]. Nevertheless, a multilaboratory study from Chaturvedi et al*.* showed a synergistic effect of posaconazole and amphotericin B against *C. glabrata* [29]. Moreover, in vitro studies demonstrated synergistic activity for the combination of isavuconazol and micafungin against *C. albicans* [28] and for the combination of micafungin with fluconazole and voriconazole against *C. glabrata* [30] supporting synergism of triazoles and echinocandins against *Candida* spp. [31]. The various results of in vitro co-treatment can be attributed to different experimental settings. Nonetheless, the correlation between laboratory studies and clinical outcome of antifungal combination therapy is not yet determined and randomized clinical studies showed that in vitro antagonism does not necessarily correlate in the clinical setting [25].

Until now, the clinical efficiency of a combination of azoles, specifically isavuconazole, and liposomal amphotericin B has not yet been sufficiently studied. In particular, clinical studies on immunocompromised patients are lacking. Some case reports have already shown a successful combination of isavuconazole and liposomal amphotericin B in disseminated mucormycosis [32, 33]. To the best of our knowledge, this is the first report illustrating successful dual antimycotic therapy with isavuconazole and liposomal amphotericin B for refractory invasive candidiasis in a liver transplant recipient. In the reported patient, this combination therapy led to clearance of *Candida* bloodstream infection and complete remission of bilomas in MRI and PET/CT imaging. Of note, this therapy was safely applicable in a patient with hepatic and renal impairment among tolerable side effects.

## Conclusion

Invasive fungal infections (IFI) in immunocompromised patients represent a very challenging situation for physicians. This report clarifies that antifungal co-treatment with isavuconazole (ISA) and liposomal amphotericin B (AMB) offers efficacy and a favorable side effect profile in a liver transplant recipient with invasive candidiasis. Antimycotic combination therapy might thus be a promising approach for persistent candidemia in immunocompromised patients. However, further investigation is needed to confirm the positive impact of dual antifungal treatment in this patient population.
